# BCL-3 Attenuation of *TNFA* Expression Involves an Incoherent Feed-Forward Loop Regulated by Chromatin Structure

**DOI:** 10.1371/journal.pone.0077015

**Published:** 2013-10-10

**Authors:** Thomas Walker, Antony Adamson, Dean A. Jackson

**Affiliations:** Faculty of Life Sciences, University of Manchester, Manchester, United Kingdom; Ludwig-Maximilians-Universität München, Germany

## Abstract

Induction of genes is rarely an isolated event; more typically occurring as part of a web of parallel interactions, or motifs, which act to refine and control gene expression. Here, we define an Incoherent Feed-forward Loop motif in which TNFα-induced NF-κB signalling activates expression of the *TNFA* gene itself and also controls synthesis of the negative regulator BCL-3. While sharing a common inductive signal, the two genes have distinct temporal expression profiles. Notably, while the *TNFA* gene promoter is primed to respond immediately to activated NF-κB in the nucleus, induction of *BCL3* expression only occurs after a time delay of about 1h. We show that this time delay is defined by remodelling of the *BCL3* gene promoter, which is required to activate gene expression, and characterise the chromatin delayed induction of *BCL3* expression using mathematical models. The models show how a delay in inhibitor production effectively uncouples the rate of response to inflammatory cues from the final magnitude of inhibition. Hence, within this regulatory motif, a delayed (incoherent) feed-forward loop together with differential rates of *TNFA* (fast) and *BCL3* (slow) mRNA turnover provide robust, pulsatile expression of TNFα . We propose that the structure of the BCL-3-dependent regulatory motif has a beneficial role in modulating expression dynamics and the inflammatory response while minimising the risk of pathological hyper-inflammation.

## Introduction

Immunological responses to perceived threats involve the coordinated action of multiple cell types over several days. Different immune cells both react to and produce pro- and anti-inflammatory cytokines to prolong and refine the immunological outcomes. Establishing the appropriate balance of cytokine expression is key to the efficacy of the immune response, as over-expression can result in hyper-inflammation and associated medical implications such as autoimmune diseases and septic shock [1]. 

In human and murine cells, the inflammatory cytokine TNFα induces transcription of its own gene product to perpetuate inflammation [2] through the NF-κB signalling pathway [3,4]. While multiple NF-κB-binding sites – κB sites - exist in the human *TNFA* promoter, the proximal κB binding (-97) confers responsiveness to LPS stimulation, whereas NF-κB bound at more distal κB sites has no significant effect on induction under this stimulus [5]. Interestingly, transcription of *TNFA* in murine macrophages is attenuated by BCL-3 [1], an IκB family member that is also induced by NF-κB. BCL-3 binds p50 and p52 homodimers and facilitates stable binding at κB sites by providing protection from ubiquitination and consequential degradation [6,7]. The effects of BCL-3 on transcription are highly context-dependent. Homodimers of p50 and p52 lack a transcription activation domain; however, this function can be provided by BCL-3 in order to induce gene transcription [6,8]. Conversely, at other promoters BCL-3 acts in a negative capacity by recruiting histone deacetylase 1 to promoters, creating a repressive chromatin state that attenuates transcription [9]. 

To date, direct regulation of *TNFA* transcription by BCL-3 has not been shown in human cell lines, although p50 homodimers have been implicated in attenuating transcription following exchange with p50/p65 at a distal κB site in the *TNFA* promoter [10]. Attenuation of LPS induced *TNFA* gene transcription in mice has also been linked to exchange of p50/65 and p50/p50 complexes [2] and while BCL-3 is not investigated in these studies, subsequent work showing a role for BCL-3 in stabilising p50/p50-DNA binding [7] is consistent with BCL-3 regulating *TNFA* transcription. Here, we characterise the induction of *TNFA* and *BCL3* gene transcription by NF-κB in the HT1080 human fibrosarcoma cell line. We provide mechanistic details to explain the rapid induction of *TNFA* gene expression and delayed expression of *BCL3*, which then acts to attenuate expression of TNFα in order to regulate the immune response. This behaviour has been recreated with a mathematical model that demonstrates the benefits of a delayed BCL-3 inhibition caused by a discrete delay in transcript production, allowing an initially rapid and large pulse of TNFα transcript production that is followed by a robust inhibition. 

## Results

### Temporal dynamics of *TNFA* and *BCL3* gene transcription

**Figure 1:**
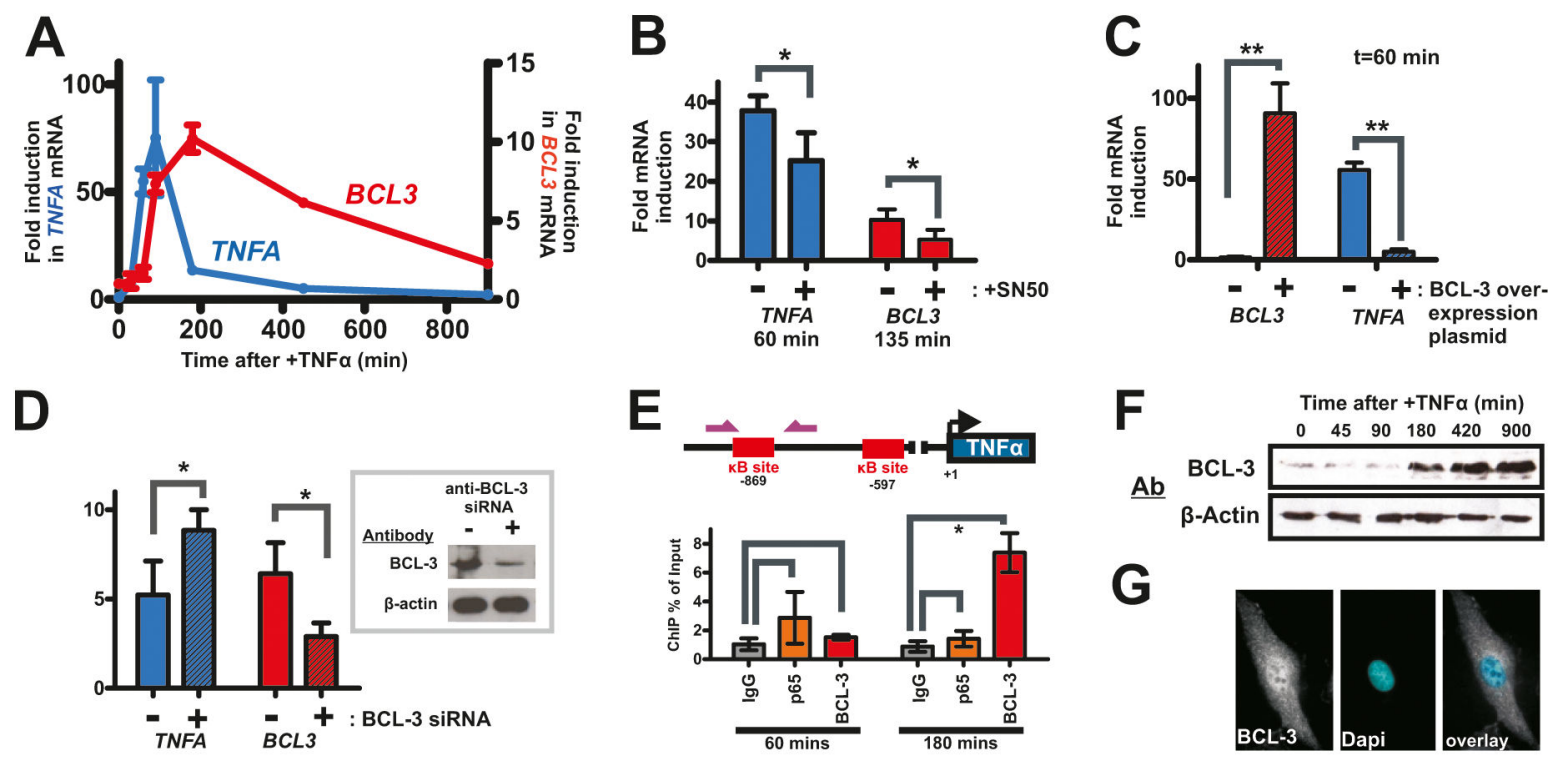
Temporal dynamics of *TNFA* and *BCL3* gene transcription in TNFα treated HT1080 cells. HT1080 cells were treated with TNFα and signalling through NF-κB monitored. Expression of *TNFA* and *BCL3* was assessed using qRT-PCR (A; n=3) to measure fold-changes in mRNA (relative to t=0) and shown to be dependent on nuclear translocation of NF-κB using the inhibitor SN50 (B; n=3). Transient over expression of BCL-3 from a constitutive promoter significantly reduced *TNFA* transcription (C; n=3) and siRNA-induced depletion of BCL-3 (inset) prior to TNFα treatment significantly increased *TNFA* transcription (D; 3h time points are shown; n=3). Temporal changes in BCL-3 occupancy at a distal (-869) κB site in the *TNFA* promoter (E; n=3) correlated with increased expression of BCL-3 (F), which though present throughout HT1080 cells was enriched in nuclei (G; 3h time-point shown). Error bars show standard deviation: *P<0.05; **P<0.01.

Stimulation of cells with the inflammatory cytokine TNFα induces transcription of multiple genes through the NF-κB signalling pathway. Following TNFα treatment, synthesis of *TNFA* and *BCL3* transcripts was measured in HT1080 cells (Figure 1A) and shown to be reduced in cells treated with an inhibitor of NF-κB nuclear movement (SN50; Figure 1B), used here under conditions where ~50% decrease in DNA binding of NF-κB is seen [11]. In cell populations treated with higher levels of SN50 much higher levels of cell death were observed (not shown). 

The sharp decrease in TNFα mRNA levels in the continued presence of stimulatory signal (Figure 1A) suggests an active mechanism for attenuating *TNFA* transcription. We confirmed this attenuation by over-expressing BCL-3 using plasmid-based transient expression [12]. After 1h stimulation with TNFα, transfected cells exhibited >60-fold increase in *BCL3* transcript levels relative to untransfected cells, with >10-fold decrease in *TNFA* transcript levels (Figure 1C). Furthermore, when RNA interference was used to inhibit *BCL3* transcription concomitant changes in *TNFA* (increased) and *BCL3* (decreased) transcription were seen (Figure 1D). To further explore the role of BCL-3 in transcriptional attenuation, we monitored the occupancy of BCL-3 and p65 at a distal κB site (-869) within the *TNFA* promoter (Figure 1E), using post-induction time points that reflect peak (60 min) and near basal (180 min) levels of *TNFA* gene transcription. A dramatic increase in BCL-3 bound at the distal κB site at the later time point correlated with a weak displacement of p65 at this site (Figure 1E). Delayed binding of BCL-3 at the *TNFA* promoter correlated with expression of BCL-3 protein (Figure 1F), which showed little increase above basal levels until ~180 min after TNFα treatment. At this time, BCL-3 was clearly, though not uniquely, localised within nuclei (Figure 1G). While BCL-3 is generally assumed to have a predominantly nuclear localisation, similar patterns have been observed in NIH 3T3 [13] and NTera-2 [8] cells.

### Delayed induction and activation of *BCL3* transcription

**Figure 2:**
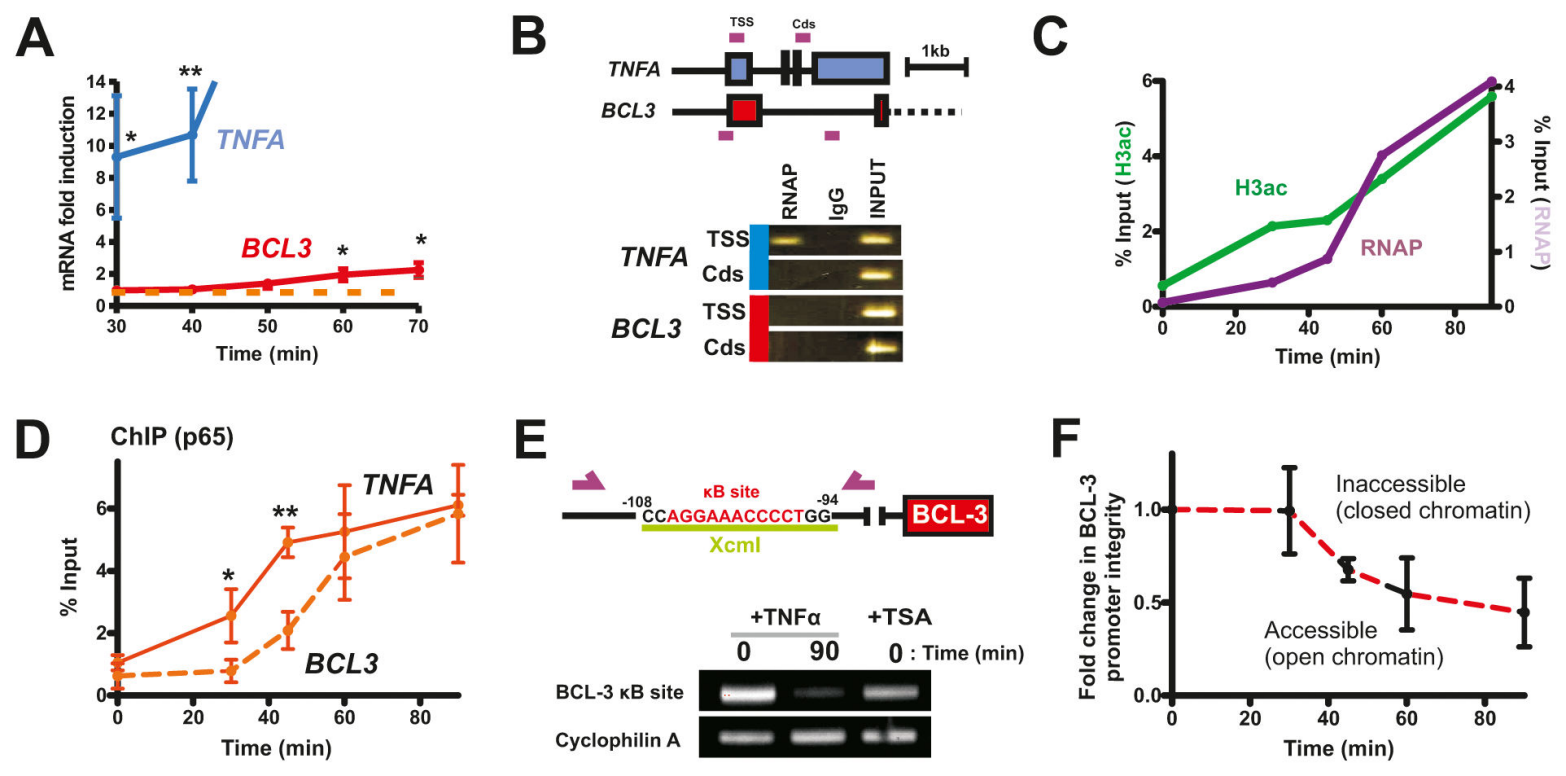
Chromatin remodelling is required for *BCL3* transcription. Following TNFα treatment of HT1080 cells clear differences in the induction dynamics of *TNFA* and *BCL3* expression were seen (A). ChIP analysis showed RNAP to be bound at the *TNFA* promoter (30) prior to TNFα treatment (B) but not within the protein coding region (Cds). At this time, RNAP at the *BCL3* promoter is not detectable and binding is clearly delayed, concomitant with a clear increase in promoter-associated histone H3 acetylation (C). Changes at the *BCL3* promoter correlated with dynamics of association of NF-κB (p56; D) and chromatin accessibility measured qualitatively (E) and quantitatively (F) by qRT-PCR. Clear changes in chromatin structure at the Xcm1 site shown were seen at 90 min following TNFα treatment and in control cells treated with TSA (400 nm for 12 h). Error bars show standard deviation: *P<0.05; **P<0.01.

Patterns of *TNFA* expression are determined by the rates of *TNFA* transcription and accumulation of BCL-3. As timing of expression of BCL-3 will define the rate of its accumulation we mapped expression at higher temporal resolution. Notably, while qPCR shows *TNFA* transcript levels to be significantly above basal levels at 30 min following TNFα treatment the equivalent increase in *BCL3* transcription was delayed until at least 60 min (Figure 2A). Using chromatin immuno-precipitation (ChIP), we established that the *TNFA* gene promoter had bound RNA polymerase II (RNAP) in unstimulated cells whereas no polymerase was present on the *BCL3* promoter (Figure 2B). However, under these conditions neither gene had RNAP within the protein coding region, implying that polymerase is bound on the *TNFA* promoter but without engaging RNA synthesis; note that this apparent promoter association reflects a steady state localisation and is also compatible with abortive cycles of transcription initiation and RNA polymerase binding at the *TNFA* promoter. Such bound but stalled RNAP allows rapid gene transcription once NF-κB signalling is induced [14,15].

The binding of RNAP at the *BCL3* TSS was assayed at 0-90 min after TNFα treatment, by which time the rate of *BCL3* transcription was greatest. Binding of RNAP increased up to this 90 min end-point (Figure 2C). A concomitant increase in acetylation of promoter associated histone H3 was also seen (Figure 2C), consistent with chromatin remodelling required for gene transcription. Cells pre-treated with the histone deacetylase inhibitor TSA, to artificially increase levels of histone acetylation, showed significantly increased levels of *BCL3* transcription at 30 min after TNFα treatment (P<0.01; Figure S1A).

### Differential binding of NF-κB at the *BCL3* and *TNFA* gene promoters

Induction of *TNFA* and *BCL3* transcription by NF-κB/p65 is dependent predominantly on promoter proximal κB sites [5,12]. Binding of p65 at TSS proximal locations was assayed by ChIP following TNFα treatment and clear temporal differences in promoter occupancy seen, with binding at the *TNFA* gene TSS occurring preferential at early times. Notably, promoter proximal binding of p65 was significantly higher for *TNFA* until ~60 min, after which time no significant differences were seen (Figure 2D). This delayed binding of p65 at the *BCL3* gene promoter correlates with the dynamics of promoter associated RNAP, acetylated histone H3 (Figure 2C) and RNA synthesis (Figure 2A) and suggests that *BCL3* transcription is dependent on chromatin remodelling.

The accessibility of chromatin to DNA binding proteins can be assayed by the relative access of restriction enzymes to DNA and resultant cutting as a surrogate reporter of chromatin structure. Using a restriction endonuclease site within the *BCL3* proximal κB site (Figure 2E) we monitored changes in the promoter structure following TNFα treatment. Nuclei were extracted from cells treated with and without TNFα, exposed to XcmI and cutting of the restriction site assessed by PCR. Following incubation with TNFα for 90 min (Figure 1A), when *BCL3* transcription is strongly induced, almost all sites were cut, and so accessible, while the target site is inaccessible in untreated cells (Figure 2E). TSA enhanced chromatin accessibility at the *BCL3* promoter (compare lanes 1 and 3 in Figure 2E) is consistent with hyperacetylation of histone and an open chromatin structure. When this assay was performed at different times following TNFα treatment and relative chromatin ‘accessibility’ determined using q-PCR a clear increase in chromatin accessibility was seen after an initial refractory period of ~30 min (Figure 2F). 

### Localisation of p65 following TNFα treatment

**Figure 3:**
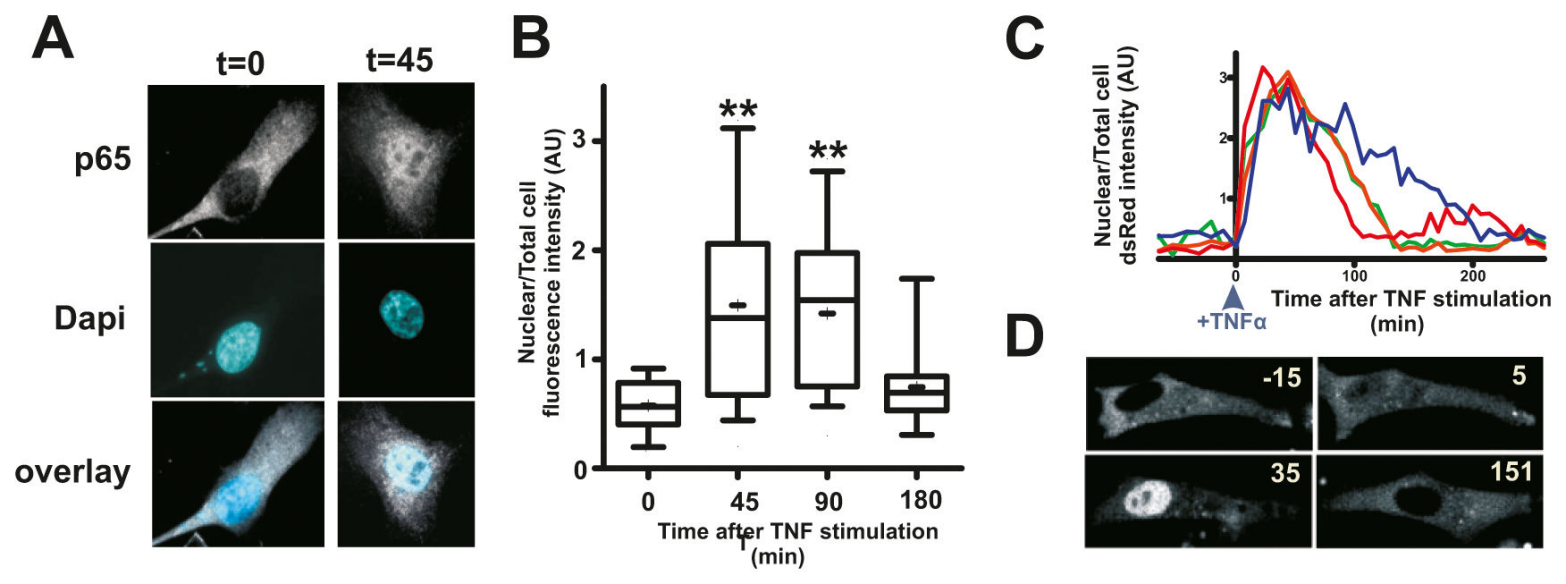
Nuclear localisation of p65/NF-κB following TNFα treatment. HT1080 cells were treated with TNFα and the distribution of NF-κB (p65 subunit) visualised by indirect immuno-flourescence (A,B) or live cell imaging of p65-dsRed (C,D) at the times shown. Time-lapse imaging shows that p65 accumulates in nuclei from 20-40 min before returning to the cytoplasm (C,D) and this is confirmed for endogenous p65 in fixed cells (B). Error bars show standard deviation: *P<0.05; **P<0.01.

Following cytokine induction, the NF-κB sub-unit p65 is released from complexes in the cytoplasm and able to move to the nucleus and activate target gene expression [4]. IκB proteins are early target genes and their expression helps to switch signalling off by returning p65 to the cytoplasm. In some cell types, if cytokine is present for long periods, p65 oscillates in and out of nuclei with a period of ~100 min [16]. Nuclear translocation of p65 was confirmed in HT1080 cells using immuno-cytochemistry, with fluorescence signal, which was initially excluded from the nucleus, showing robust nuclear accumulation from 45-90 min (Figure 3A and B; P<0.01). At later time points the nuclear p65 signal was no longer significantly greater than that in unstimulated cells (Figure 3A and B; P>0.05). As the rate at which p65 enters the nucleus and the duration of its occupancy will dictate the transcriptional responses of target genes, we also monitored HT1080 cells expressing p65-dsRed by time-lapse microscopy. Following TNFα-activated NF-κB signalling, p65 quickly became nuclear, with almost all dsRed-p65 occupying the nucleus at ~30-50 min, before returning to the cytoplasm at ~100 min (Figure 3C and D and S2A). This data defines the time window during which NF-κB target genes will be exposed to optimal levels of nuclear-localised transcription factor.

### Modelling the induced BCL-3 inhibition of *TNFA* transcription

**Figure 4:**
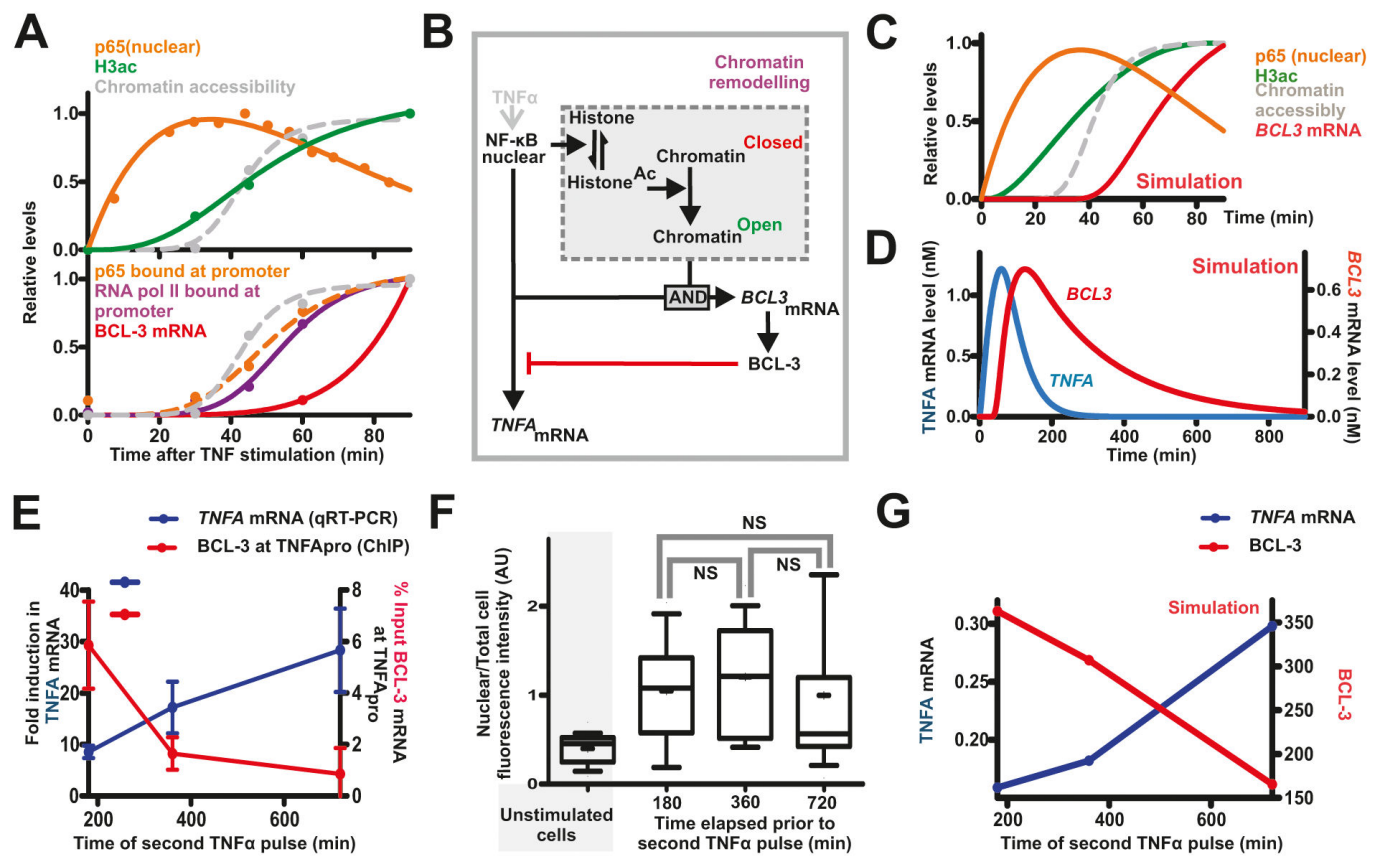
Modelling TNFα induced transcription at the *BCL3* and *TNFA* promoters. A model linking *TNFA* and *BCL3* expression shows how the timing of events that regulate *BCL3* transcription (A; data from Figures 1and 2) can be recapitulated using an ODE model (B; Information S2 for parameters) that mimics the data in simulations (C,D). During pulsatile stimulation, the separation of two TNFα treatments only weakly affects the nuclear localisation of NF-κB (E; p65 as in Figure 3B) while dramatically influencing the time-dependent expression of *TNFA*, as a result of promoter-bound BCL-3 (F). This behaviour is reproduced by the model (G).

To assess the effect of delayed *BCL3* transcription on *TNFA* transcription, we created an ODE model for transcriptional regulation of these genes that also incorporates chromatin remodelling events (Supporting Information S1). Relative levels of nuclear p65, acetylated H3, chromatin accessibility, p65 bound at the *BCL3* gene promoter, RNAP bound at the *BCL3* gene promoter and *BCL3* mRNA levels are shown over 90 minutes following TNFα stimulation; a value of 0 is assigned at t=0 and 1 assigned to the maximal average value occurring in this time frame, with all other values expressed as a fraction of the maximal value (Figure 4A). Acetylated H3 increased, after a delay, following p65 nuclear translocation and this was followed, in turn, by elevated chromatin accessibility, promoter associated p65, RNAP and finally mRNA synthesis (Figure 4A). A representation of the processes involved in NF-κB-induced *BCL3* transcription is shown in Figure 4B. Rates of histone acetylation, chromatin ‘opening’ and facilitated *BCL3* transcription are assumed to be dependent on levels of nuclear NF-κB, histone acetylation and chromatin accessibility, respectively. The rates used (Supporting Information S2) were fitted (Figure 4C) to experimentally observed rates (Figure 4A) and the modelled induction of *BCL3* transcription placed within the context of subsequent protein production and inhibition of *TNFA* transcription (Figure 4B). Simulated outputs of *TNFA* and *BCL3* transcription (Figure 4D) show a strong, qualitative correspondence to the patterns of synthesis observed experimentally (Figure 1A). 

### BCL-3 attenuates *TNFA* transcription

During a natural inflammatory response cytokine signalling is likely to be pulsed rather than continuous, based on local changes in cytokine concentration. This raises questions about the role of BCL-3 in attenuating *TNFA* transcription at different stages of the inflammatory response. To address this, cells were stimulated with TNFα for 180 minutes (by which time *TNFA* transcription attenuates; Figure 1A), washed to remove the cytokine and subsequently grown for 180, 360 and 720 min, before BCL-3 bound at the *TNFA* promoter was measured (Figure 4E; right axis). In parallel cultures (Figure S2B and C), cells were treated with a second 60 min pulse of TNFα and induction of *TNFA* transcription measured (Figure 4E; left axis). Consistent with BCL-3 attenuating *TNFA* transcription, high levels of *TNFA* transcription correlated with increased separation of the TNFα pulses, with longer pulse separations correlating with reduced BCL-3 bound at the *TNFA* promoter (Figure 4E). In this scenario, the observed differences in *TNFA* transcription arise despite similarities in nuclear NF-κB levels following the second TNFα treatment (Figure 4F), consistent with promoter accessibility and not transcription factor concentration being a key determinant of TNF synthesis. Our model that incorporates a chromatin-based time delay in BCL-3 expression recreates this behaviour (compare simulation Figure 4G with data Figure 4E; Figure S2D). These experiments show that when signalling through NF-κB is activated by TNFα the elapse time between consecutive pulses can have a profound influence on temporal levels and patterns of TNF synthesis, which is dependent on the amount of BCL-3 bound to the *TNFA* promoter at the time of induction. 

### Delayed BCL-3 production regulates *TNFA* transcription kinetics

**Figure 5:**
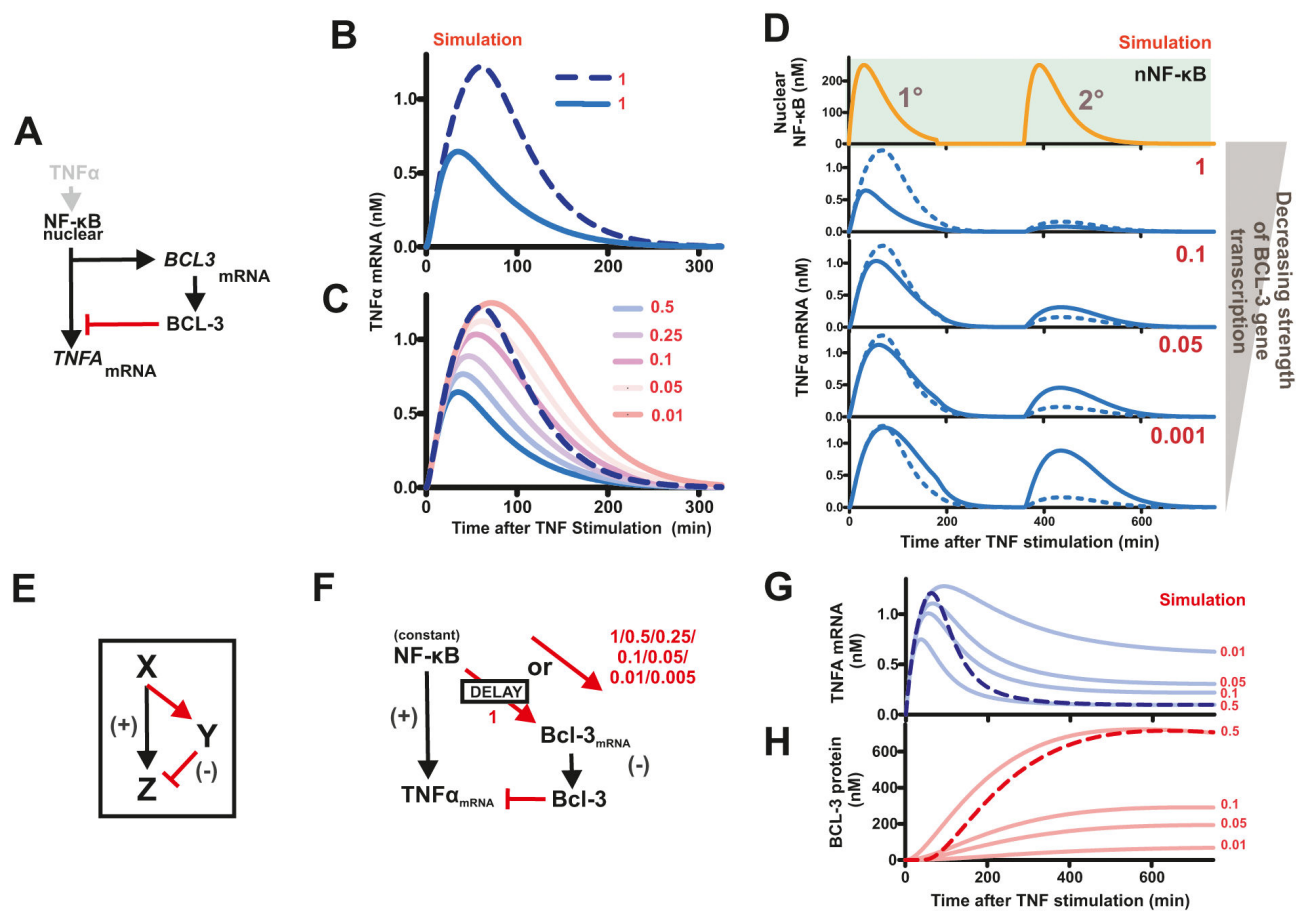
Modelling the influence of delayed *BCL3* expression on *TNFA* transcription. The ODE model shown in Figure 4B was simplified by removing the chromatin remodelling step (A) to simulate how this delay influences *TNFA* transcription (B-D). At different times following TNFα stimulation, the output from this model (B, solid line) is lower than with the time-delayed model (B, dashed line), though differences can be partly recapitulated simply by reducing expression of BCL-3, when both a single (C) or double (D) pulse of TNFα is used. This model motif can be represented by an Incoherent Feed-forward Loop (E), to test a simplified version of the *TNFA* transcription/BCL-3 model with time-delayed or continuous induction (with decreasing magnitude, red numbers) of *BCL3* transcription (F). Simulations for *TNFA* mRNA (G) and BCL-3 protein (H) are shown (solid lines) and compared to the chromatin delay model (dashed lines).

A model capable of recreating patterns of *BCL-3/TNFA* transcription provides a tool to assess the functional significance of the differential response times of *BCL3* and *TNFA* transcription induced by NF-κB. To asses the impact of the delayed BCL-3 expression biologically, we used a simplified ‘non-delay model’ in which *BCL3* and *TNFΑ* transcription were activated together in response to nuclear NF-κB (Figure 5A). In these simulations, we also compared how changes in TNFα mRNA predicted by the time-delayed model – and validated experimentally – corresponded with models in which the concentration of BCL-3 was altered but without invoking a time delay. The rapid induction of BCL-3 synthesis in the simplified non-delay model quickly attenuates *TNFA* transcription, resulting in dramatically reduced cytokine expression in comparison to the delay model (Figure 5B). As expected, in the simplified model, decreasing the rate of *BCL3* gene transcription (Supporting Information S2, parameter k112) was seen to correlate with an increase in the initial *TNFA* transcript pulse (Figure 5C). When different rates of *BCL3* transcription were simulated, it was necessary to reduce the rate 100-fold in order to produce levels of *TNFA* expression comparable to those seen in the time-delayed model (Figure 5C). As expected for a transcriptional suppressor, these simulation show that manipulation of BCL-3 concentration is able to modulate *TNFA* transcription. However, while substantially reducing *BCL3* transcription can partially mimic *TNFA* transcription seen for the time-delayed model, this behaviour is restricted to changes in gene expression that result from a single cycle of NF-κB translocation. Notably, when double (or multiple) pulses of signalling through NF-κB are induced the patterns of *TNFA* transcription seen for the non-delay model are clearly different to those for the time-delay model (Figure 5D), with the latter corresponding to the experimental data (Figure 4). Hence, delaying transcription of *BCL3*, relative to *TNFA*, gives a high level of TNFα in the primary response, which is then attenuated by BCL-3-induced inhibition of *TNFA* gene expression. Such a genetic circuit allows cells to react efficiently to local inflammatory cues in order to propagate potentially small or transitory signals but crucially limits the duration of the primary response. 

### Architecture of I-FFL motif structures that generate distinctive output kinetics

When a single activating factor drives both positive and negative influences on the same output an incoherent feed-forward loop (I-FFL) is generated (Figure 5E). Such motifs have previously been shown to create pulse-like transcriptional responses [17]. In this study, we addressed how altering the timing of the activating and suppressing inputs of such a FFL defines its output. In our example, the activating input (NF-κB→*TNFA* transcription) responds more rapidly than the inhibitory input (NF-κB→BCL-3, which inhibits *TNFA* transcription) based on a refractory period that delays synthesis of *BCL3* transcripts. This delay represents the time required to remodel chromatin at the *BCL3* promoter, a process which we show is an essential prerequisite for transcription complex assembly (Figure 3).

A simplified model of BCL-3/*TNFA* interactions (Figure 5F), in which nuclear NF-κB levels are constant (200nM) rather than transitory, allows induced model components to reach stable steady state conditions (other than zero). In this model, *BCL3* transcription is either delayed by chromatin remodelling or induced without delay in response to nuclear NF-κB, but with different rates of transcription (Parameter k112; range 0.005-1). Without delay, low rates of *BCL3* transcription result in an initial peak of *TNFA* transcription but with a high stable steady state (Figure 5G). This reflects the low steady state expression of BCL-3 (Figure 5H) and consequent weak attenuation of *TNFA* transcription. In contrast, delayed production of BCL-3 (Figure 5H – dashed line) mimics experimental data, with an immediate peak of expression and strong attenuation at later times producing distinctive ‘shark’s fin’ *TNFA* transcription kinetics (Figure 5G – dashed line). The characteristic pulse-like response of an I-FFL is, therefore, defined by the induction kinetics of the inhibitor. In this example, the delay in inhibitor production effectively uncouples the response rate and the final magnitude of inhibition. This pattern of control is likely to have important implications during an immune response, where it is necessary to mount a rapid initial response that is subsequently attenuated in order to limit pathological side-effects. 

## Discussion

Mammals respond to insults such as infection or injury by activating an inflammatory response. Signalling by NF-κB is a key regulator of this process. NF-κB target genes - ~500 are known - exhibit differential temporal expression, with ‘early’, ‘mid’ and ‘late’ response genes contributing to timing of the immune response following induction [18]. Delayed induction has been linked to promoter function, so that NF-κB binding occurs initially at constitutively accessible promoters and is delayed at promoters that only become accessible after chromatin remodelling [19]. As temporal regulation is an innate feature of the immune response, we explored how expression of the pro-inflammatory cytokine TNFα can be attenuated to control the extent of inflammation.

### Differential regulation of *TNFA* and *BCL3* expression

In this study, we show that the transcription of two genes induced by NF-κB is initiated at different times and demonstrate, using mathematical modelling, how this differential induction can have a profound effect on gene expression. *TNFA* is an early NF-κB target gene [18], which is regulated by the IκB family protein BCL-3 [1]. *TNFA* and *BCL3* display distinct response profiles following signalling, with a rapid induction in *TNFA* transcription and delayed transcription of *BCL3* (Figure 1). To understand the temporal regulation of *TNFA* expression, we showed that the *TNFA* promoter is constitutively primed for RNA synthesis whereas chromatin remodelling of the *BCL3* promoter is required for NF-κB binding (Figure 2). With this circuitry, an initial but transient burst of *TNFA* expression is attenuated once BCL-3 begins to accumulate (Figure 2). The expression of these genes was shown to be dictated by the chromatin state at their promoters and consequent accessibility of transcription factors and RNAP to DNA (Figure 2). Chromatin remodelling is a multi-step process [20,21], which involves processes such as histone acetylation, recruitment of remodelling complexes, nucleosome re-positioning and pre-initiation complex formation. At promoters, changes in chromatin structure can significantly impact on the timing of gene expression [19]. *TNFA* transcription rapidly responds to TNFα-induced NF-κB signalling (Figure 1). However, we have shown that a major negative regulator of *TNFA* transcription, BCL-3, exhibits far slower transcriptional induction (Figure 2), which required chromatin remodelling before NF-κB is able to bind at the promoter of this gene. This behaviour has been recreated with a mathematical model using experimentally verified parameter values (Supporting Information S2). Model simulations demonstrated the biological importance of time-delayed BCL-3 synthesis as a means of regulating TNFα output (Figure 4 and 5), so that a rapid initial synthesis of TNFα is coupled to robust subsequent inhibition. 

### Delayed expression of BCL3 involves chromatin remodelling

By delaying expression of inhibitory BCL-3, the time taken to express sufficient protein to inhibit *TNFA* transcription can be uncoupled from the magnitude of the inflammatory response. Hence, rapid and sizeable initial pulses of *TNFA* mRNA are permitted before a robust later inhibition of transcription (mediated by BCL-3) is seen. Cells are consequently able to produce relatively large but strictly transient bursts of cytokine in response to an initial stimulation. Using simulations in which transcription of *TNFA* and *BCL3* were activated simultaneously, changes in the maximal *BCL-3* transcription rate (parameter k112) can recreate the initial *TNFA* pulse size when the maximal promoter rate is decreased to 1/100^th^ of that used in the chromatin delay model (Figure 5B). However, while initial *TNFA* transcript levels are comparable to levels in the delayed transcription model, down-regulation of *TNFA* transcription as a response to subsequent NF-κB signalling is less pronounced – resulting in high, persistent levels of *TNFA* mRNAs (Figure 5D). Such a scenario would lead to excessive cytokine signalling and could result in associated inflammatory disorders. 

Chromatin structure is well known to contribute to the hierarchy of genomic features that control eukaryotic gene expression. Different classes of gene promoters with different characteristic patterns of transcription initiation have been described [22] and the ability of some promoters to occupy transiently active or inactive states contributes to the innate stochasticity of expression [23,24]. During inflammation, activated NF-κB must access binding sites in gene promoters to drive target gene expression. In some cases promoters are constitutively open [25] and these may be primed with RNA polymerase prior to induction (Figure 2). In other situations changes in chromatin structure represent a rate-limiting step that regulates expression of key genes based on cell lineage [26]; IL-4 expression in Th2 lymphocyte cells was shown to involve a stochastic chromatin remodelling step such that only a fraction of the Th2 cell population were see to express IL-4 following antigen stimulation. Such cell type-dependent chromatin effects are likely to be extremely important in generating heterogeneity in a physiological setting [27]. The *BCL3* promoter analysed in our in vitro study is shown to be inaccessible to RNA polymerase at the time of TNFα addition so that remodelling of the promoter chromatin must precede activation of *BCL3* transcription. While we show, based on chromatin accessibility (Figure 2), that chromatin remodelling within the BCL3 promoter occurs at a cell population level over a period of 30-90 minutes post TNFα treatment, analysis of the molecular complexities of this process was beyond the scope of our study. 

### Regulatory principles of an incoherent feed-forward loop

When a single activating factor has both positive and negative regulatory influences on the same output event an incoherent feed-forward loop is formed (I-FFL – a schematic is shown in Figure 5E). Such motifs have previously been shown to create pulse-like transcriptional responses [17]. In this study we have addressed the issue of relative timing in the two legs (- and +; relating to their effect on the production of output) of such a feed-forward loop. We have shown how the positive leg of the feed-forward loop (NF-κB→*TNFA* transcription) responds more rapidly than the inhibitory leg (NF-κB→BCL-3 which inhibits *TNFA* transcription) which, as result of chromatin remodelling events required to produce *BCL3* mRNA, experiences a delay before acting. 

A simplified model of BCL-3/*TNFA* interactions in which nuclear NF-κB levels are constant (200 nM), rather than transitory, allows induced model components to reach stable steady state conditions (other than zero) (Figure 5F). In this scenario, the transcriptional induction of *BCL3* occurs either via a chromatin mediated delay or immediately, but with varied levels of magnitude (from 1 to 0.005; Figure 5F). Reducing the maximal transcription rate of the *BCL3* gene clearly causes an increase in the initial *TNFA* mRNA peak but also occurs with an associated higher stable steady state (Figure 5G); this behaviour is explained by the BCL-3 protein levels produced. Lower *BCL3* transcription rates reduce rates of BCL-3 protein production but also reduce the final steady state of protein accumulation (Figure 5H). As a result, inhibition of *TNFA* transcription is reduced so that an increase in steady state mRNA levels is seen. In contrast, delayed production of BCL-3 (Figure 5H) produces a robust initial expression of *TNFA* but efficient inhibition at later times. 

In conclusion, our data describe the characteristics pulse-like output of an I-FFL in which the robust inflammatory response defined by expression of TNFα is subsequently switched off by BCL-3. Both *TNFA* and *BCL3* promoters are controlled by NF-κB, with a chromatin-dependent delay in expression of BCL-3 defining the duration of optimal TNFα synthesis. In a recent study, Buetti-Dinh et al. showed how a non-monotonous response (i.e. a bell shape response at steady state, which is equivalent to pulse generation during a transient response) characterises the control of gene expression using multiplicative interactions between activators and inhibitors [28]. Notably, while pulsatile behaviour was shown to be a robust, innate feature of feed-forward loops, the pulse peak is reflected by the combination of parameter values, with a relatively flat response seen for many parameter combinations. In comparison, our analysis emphasises how uncoupling the inhibitory component of the feed-forward loop with a time delay serves to enhance the pulsing behaviour. In addition, in the I-FFL described herein, the different rates of turnover of *TNFA* and *BCL3* mRNAs (Supporting Figure S1B and Supporting Information S2) accentuate the rapid increase and subsequent inhibition of TNFα synthesis. These observations emphasise how timing contributes to the flow of information through genetic network, with features such as chromatin remodelling and mRNA turnover acting as discrete temporal regulators to control gene expression.

## Materials and Methods

### Cell line and stimulation

HT1080 cells (ATCC, LGC Standards, Teddington, Middlesex, UK) were grown in DMEM media (Invitrogen Gibco^TM^ Ltd., Paisley, UK) supplemented with 10% FBS and 1% NEAA at 37°C with 5% CO_2_ and stimulated with a saturating dose (10 ng/ml) of human recombinant TNFα (Merck, Darmstadt, Germany) unless otherwise stated. As required, cells were treated with SN50 (30 μg/ml for 1 h before TNFα stimulation; Merck, Darmstadt, Germany) or trichostatin A (TSA; for 12 hours prior to TNFα stimulation using concentrations shown; Sigma-Aldrich Company Ltd., Dorset, UK). Rates of mRNA decay were measured by inhibiting transcription with actinomycin D (0.2 μg/ml; Sigma-Aldrich Company Ltd., Dorset, UK). 

### qRT-PCR

RNA was extracted using QiaShredder homogenisers and RNAeasy kit (Qiagen Ltd., Crawley, UK) and converted to cDNA with High Capacity cDNA Reverse Transcription Kit (Applied Biosystems Ltd., Warrington, UK). RT-PCR was preformed with an Applied Biosystems 7300 Real Time PCR System (Applied Biosystems Ltd., Warrington, UK), ABI Power SYBR® Green PCR Master Mix and using primer sets (Applied Biosystems Ltd., Warrington, UK): TNFα-FOR (CTCTTCTGCCTGCTGCACTT), TNFα-REV (GCTGGTTATCTCTCAGCTCCA); BCL-3-FOR (CCCTATACCCCATGATGTGC), BCL-3-REV (GGTGTCTGCCGTAGGTTGTT) and Cyclophilin A primers [16]. All primers were obtained from Sigma-Aldrich Company Ltd., Dorset, UK. 

### Western blots

Whole cell protein extracts (10 ng/lane) were used in Western blot. BCL-3 was detected with a Rabbit polyclonal primary antibody (sc-185; Santa Cruz Biotechnology, Heidelberg Germany) and Goat Anti-Rabbit IgG (H+L)-HRP Conjugate (Cat # 172-1019; Bio-Rad Laboratories) secondary antibody. β-actin was detected with mouse monoclonal antibody (A1978; Sigma-Aldrich, Dorset, UK) and Goat Anti-Mouse IgG (H+L)-HRP Conjugate (Cat # 172-1011; Bio-Rad Laboratories, Hemel Hempstead, UK) secondary antibody.

### Molecular biology and Microscopy

BCL-3 over-expression vector was obtained from Allan Brasier’s Laboratory [12] and the p65dsRed plasmid has been previously described [16]. Transfection was performed using ExGen500 (Fermentas, St Leon-Rot, Germany) following the routine procedure. Samples were analysed 48 h following transfection. For time-lapse imaging, cells were grown on 35 mm tissue culture dishes (Iwaki, Japan) in 3 ml of media, visualised using a Zeiss LSM 710 confocal laser scanning microscope and nuclear/total fluorescence levels determined using CellTracker software version 0.6 [29].

For immuno-labelling, cells were grown on cover slips (22 mm diameter; Scientific Laboratory Supplies Ltd, Wilford, UK) and fixed with 4% paraformaldehyde (10 min; Electron Microscopy Sciences). After rinsing in PBS, samples were washed with 1% Triton X-100 in PBS (15 min; Sigma-Aldrich), washed 3x with PBS and 3x with PBS+ (PBS, 0.1% Tween-20, 1% BSA). Cover slips were incubated with PBS+ (30 min) prior to incubation with p65 binding antibody (1:500 in PBS+; 4°C for 16 h; #3034; Cell Signalling Technology). Cover slips were washed 3x in PBS and 3x in PBS+ and incubated with Cy^TM^3-conjugated AffiniPure Donkey Anti-Rabbit IgG (H+L) secondary antibody (1:1000 in PBS+; 30 min; Jackson Immuno Research Laboratories, Inc.). Cover slips were finally washed 3x in PBS+, 3x in PBS and mounted in Vectorshield containing DAPI (Vector Labs). Images were collected using Zeiss LSM 710 confocal laser scanning microscope. 

### ChIP

Analysis by ChIP was performed as described [30]. Chromatin was sonicated using a Cole Palmer Ultrasonic processor. Primers for amplifying at the distal *TNFA* promoter κB site (Figure 1E) were Distal κB site-FOR (GGCTCTGAGGAATGGGTTAC) and Distal κB site- REV (GAGGTCCTGGAGGCTCTTTC). Primers amplifying the *TNFA* gene TSS were TNF TSS-FOR (GGACAGCAGAGGACCAGCTA) and TNF TSS-REV (GTCCTTTCCAGGGGAGAGAG). Primers amplifying the *BCL-3* gene TSS were BCL-3 TSS-FOR (GGGCCAGAAAGACAAAAACA) and BCL-3 TSS-REV (CCCAGGGGTTTCCTGGAC). Antobodies used are as follows: Anti-mouse IgG (12-371), Anti-p65 C terminus (06-418), Anti-RNA polymerase II (050623), anti-acetyl histone 3 (06-599, all Millipore) and anti-BCL-3 (sc-185; Santa Cruz Biotechnology).

### Chromatin accessibility assay

Cells were stimulated with TNFα, trypsinised, spun down, washed 2x with cold PBS, cell membranes were then lysed with a Cell Lysis buffer (5 mM PIPES pH 8.0; 85 mM KCl; 0.5% Nonidet P-40). Nuclei were re-suspended in NE Buffer 2 (New England BioLabs Ltd., Hitchin, UK) and incubated with XcmI enzyme (New England BioLabs Ltd., Hitchin, UK; 37°C; 1 h), the reaction was then terminated (65°C; 30 min), followed by genomic DNA extraction. DNA was quantified in a qRT-PCR reaction (10 min at 95°C; 40 cycles - 95°C 15 seconds/58°C 1 min) with the primers XcmIsite-FOR (GGGCCAGAAAGACAAAAACA) and XcmIsite-REV (CCACTCACCGGGGTAGTAAA).

### Data analysis modelling and graphical representation

Model data was generated using MATLAB R2010a (MathWorks) and data was plotted using GraphPad Prism 5 (GraphPad Software, Inc). P values were calculated using Students T test, assuming a normal distribution of data. Error bars show standard deviation.

## Supporting Information

Figure S1 (A) Pre-treatment of HT1080 cells with 400 nM TSA prior to induction caused a significant induction in the level of BCL3 mRNA response to 30 minutes of TNFα, in contrast to 200 nM TSA pre-treatment (n=3). *P<0.05; **P<0.01. (B) Half lives of *TNFA* and BCL3 transcripts from cells stimulated with TNFα, treated with a transcription inhibitor and then left for increasing lengths of time (x axis). Relative levels of transcript are in comparison to cells at t=0 mins following stimulation with 60 minutes of TNFα. Half lives of the transcripts are calculated at the time point at which transcript levels have degraded to half initial values (red dashed line). (C) Protein half lives are determined from data in Keutgens et al. [31]. One phase decay lines are fitted; R^2^ values are BCL3 mRNA = 0.961; *TNFA* mRNA = 0.957; BCL-3 protein = 0.913.
(TIF)

Figure S2 **Time course of 15 cells expressing p65-dsRed following stimulation with TNFα at t=0; as in Figure 3C,D.** Numbers relate to individual cells analysed. Stimulation of HT1080 cells with secondary NF-κB stimuli: (B) Schematic of experimental protocol used to provide secondary TNFα stimuli to HT1080 cells previously stimulated with a 180 minute TNFα pulse. Cells were washed twice with PBS following primary stimulation and left for 180, 360 or 720 minutes in the absence of TNFα; at which point either BCL-3 bound at the *TNFA* promoter was determined by ChIP (as before – Figure 1E) or cells were stimulated again with TNFα for a further 60 minutes and induction levels measured by qRT-PCR (see Figure 4E). (C) Nuclear NF-κB stimuli profiles used in simulations to represent secondary TNFα stimuli and (D) output profiles of *TNFA* mRNA produced by such stimuli profiles.
(TIF)

Supporting Information S1 Information S1 and S2 describe the parameters and protocols used during modelling. (DOC)

Supporting Information S2 Information S1 and S2 describe the parameters and protocols used during modelling. (DOC)
