# Supplementary figures and images for: BCL-3 Attenuation of TNFA Expression Involves an Incoherent Feed-Forward Loop Regulated by Chromatin Structure
Source: PLoS One. 2013 Oct 10;8(10):e77015. doi: 10.1371/journal.pone.0077015 (PMC3794926; doi:10.1371/journal.pone.0077015)

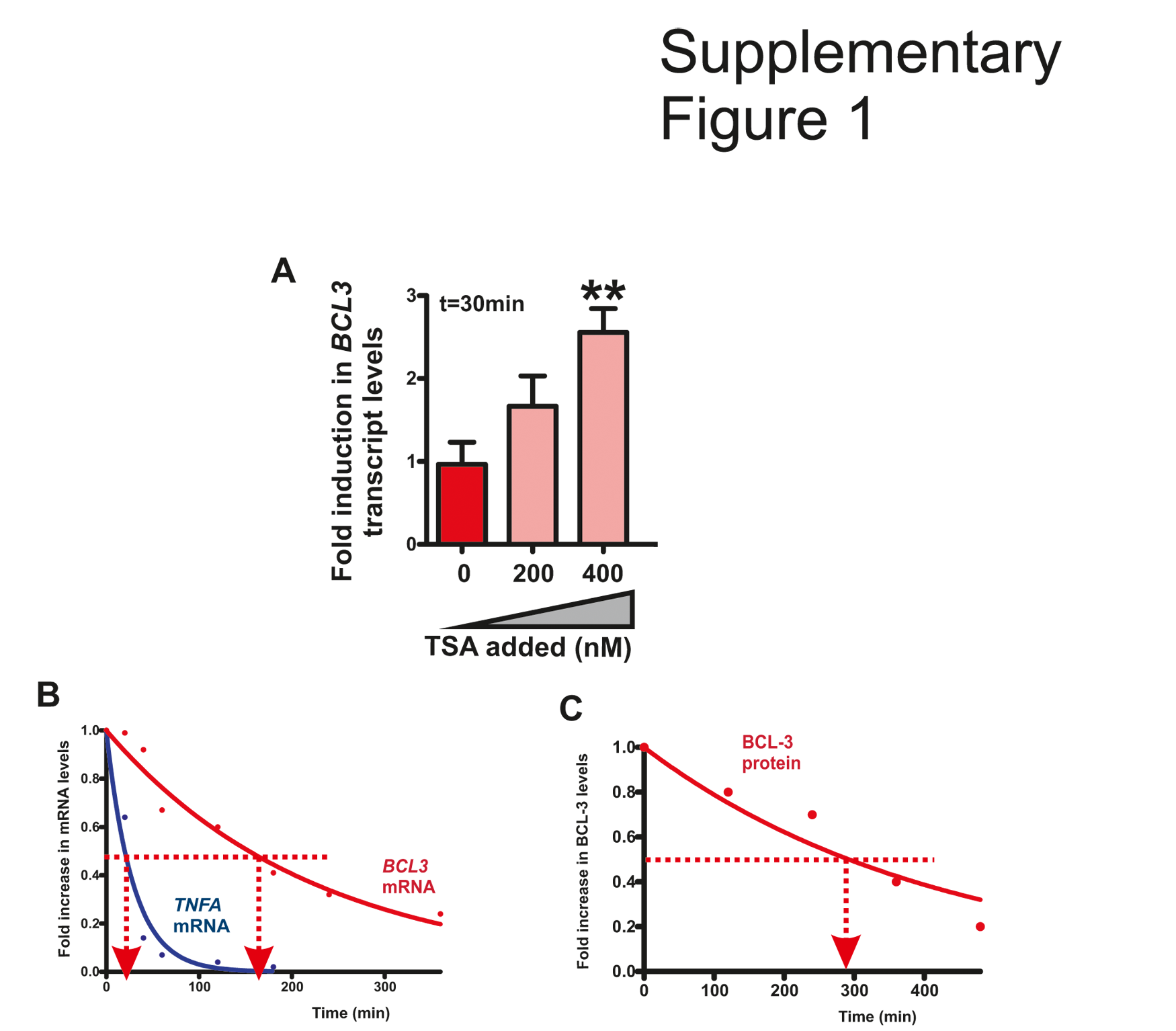

Supplement: Figure S1 — (A) Pre-treatment of HT1080 cells with 400 nM TSA prior to induction caused a significant induction in the level of BCL3 mRNA response to 30 minutes of TNFα, in contrast to 200 nM TSA pre-treatment (n=3). *P<0.05; **P<0.01. (B) Half lives of TNFA and BCL3 transcripts from cells stimulated with TNFα, treated with a transcription inhibitor and then left for increasing lengths of time (x axis). Relative levels of transcript are in comparison to cells at t=0 mins following stimulation with 60 minutes of TNFα. Half lives of the transcripts are calculated at the time point at which transcript levels have degraded to half initial values (red dashed line). (C) Protein half lives are determined from data in Keutgens et al. [31]. One phase decay lines are fitted; R2 values are BCL3 mRNA = 0.961; TNFA mRNA = 0.957; BCL-3 protein = 0.913. (TIF) [file pone.0077015.s001.tif]

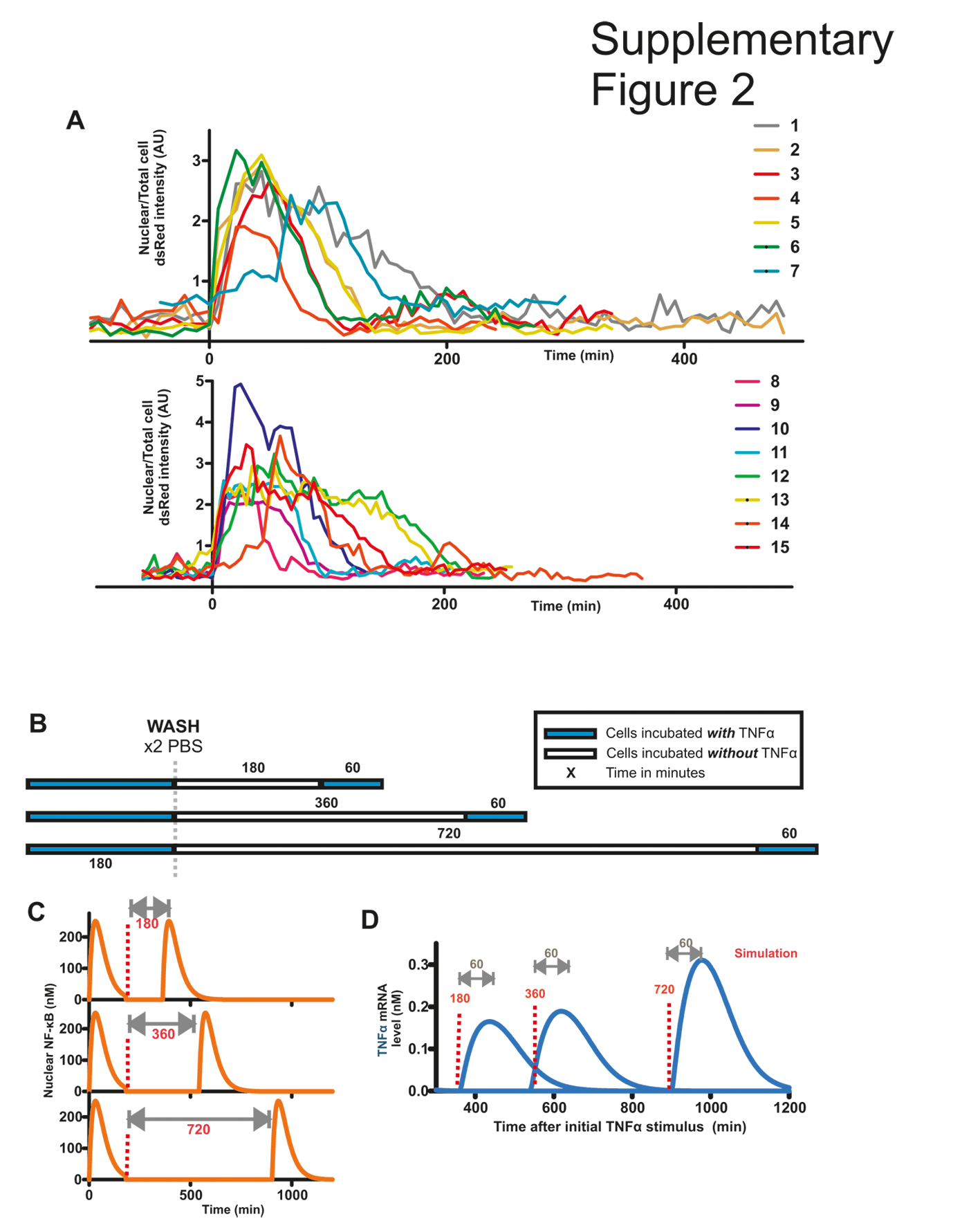

Supplement: Figure S2 — Time course of 15 cells expressing p65-dsRed following stimulation with TNFα at t=0; as in Figure 3C,D. Numbers relate to individual cells analysed. Stimulation of HT1080 cells with secondary NF-κB stimuli: (B) Schematic of experimental protocol used to provide secondary TNFα stimuli to HT1080 cells previously stimulated with a 180 minute TNFα pulse. Cells were washed twice with PBS following primary stimulation and left for 180, 360 or 720 minutes in the absence of TNFα; at which point either BCL-3 bound at the TNFA promoter was determined by ChIP (as before – Figure 1E) or cells were stimulated again with TNFα for a further 60 minutes and induction levels measured by qRT-PCR (see Figure 4E). (C) Nuclear NF-κB stimuli profiles used in simulations to represent secondary TNFα stimuli and (D) output profiles of TNFA mRNA produced by such stimuli profiles. (TIF) [file pone.0077015.s002.tif]
