# Supplementary material for: BCL-3 Attenuation of TNFA Expression Involves an Incoherent Feed-Forward Loop Regulated by Chromatin Structure
Source: PLoS One. 2013 Oct 10;8(10):e77015. doi: 10.1371/journal.pone.0077015 (PMC3794926; doi:10.1371/journal.pone.0077015)
Supplement: Supporting Information S1 — (DOC) [file pone.0077015.s003.doc]

**Supplementary Information S1**

**Lines fitted to relative experimental time courses (Figure 4A)**

*Equations used (t=time);*

**Two phase exponential association:** Y=Y_MAX1_(1-e^(-k1*t)^)+Y_MAX2_(1-e^(-k2*t)^);

**Plateau followed by one phase association:**Y=Y_0_ when t<t_x_;

Y= Y_0_+(P- Y_0_)*(1-e^(-k*(t-tx))^) when t>t_x_.

**One site – specific binding with Hill slope:** Y=Y_MAX_*t^H^/(K_d_^H^+t^H^)

**Nuclear levels of p65/NF-κB (orange line)**

The induced nuclear localisation of NF-κB following TNFα stimulation had previously been measured using single cell fluorescence microscopy of cells expressing p65-dsRed (Figure 3C and D). Data from 15 cells analysed (individual traces shown in Supplementary Figure S2A) was averaged; with the average nuclear/total cell fluorescence at t=0 taken as basal level (i.e. equal to 0) and increases above this value expressed as a fraction of the largest average increase seen for all cells. A Two Phase exponential association curve was fitted to the data (R^2^=0.9857) and used to define an equation which plots levels of nuclear p65 as a function of time:


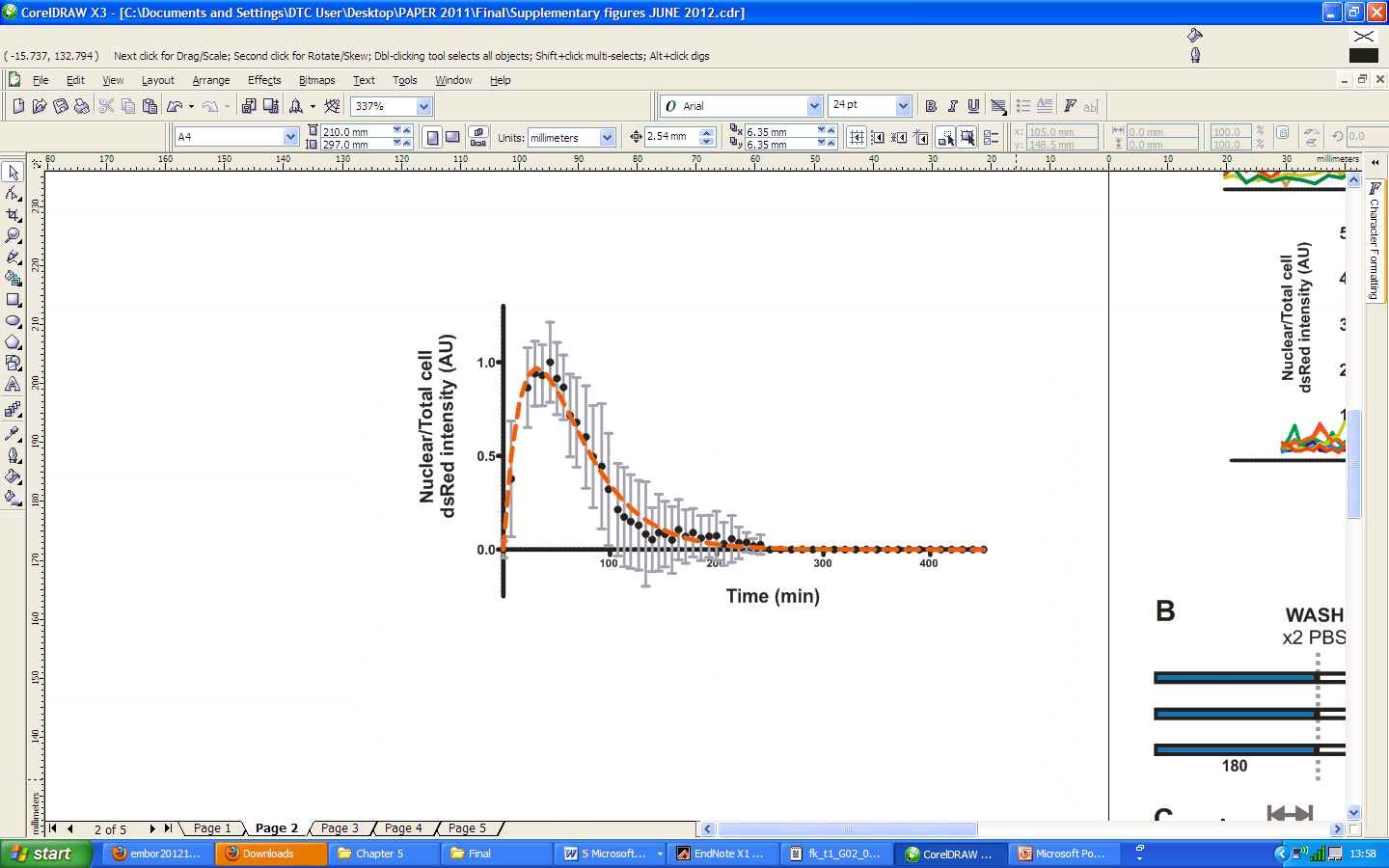


Two phase exponential association, R^2^=0.986

Y_MAX1_=144; k1=0.03218; Y_MAX2_=144; k2=0.0316;

**Histone 3 acetylation levels (green line) – from Figure 2C.**

One site – specific binding with Hill slope, R^2^=0.996

Y_MAX_=1.19; H= 2.872; K_d_=49.64.

**Chromatin accessibility (grey dashed line)**

A time course of increased digestion of an XcmI site within the proximal κB site of the *BCL3* gene promoter is shown in Figure 2G. To measure this as a function of increasing chromatin accessibility (rather than decreasing DNA template), the graph is inverted (i.e. each value is subtracted from 1) and re-plotted, with an appropriate curve fitted:


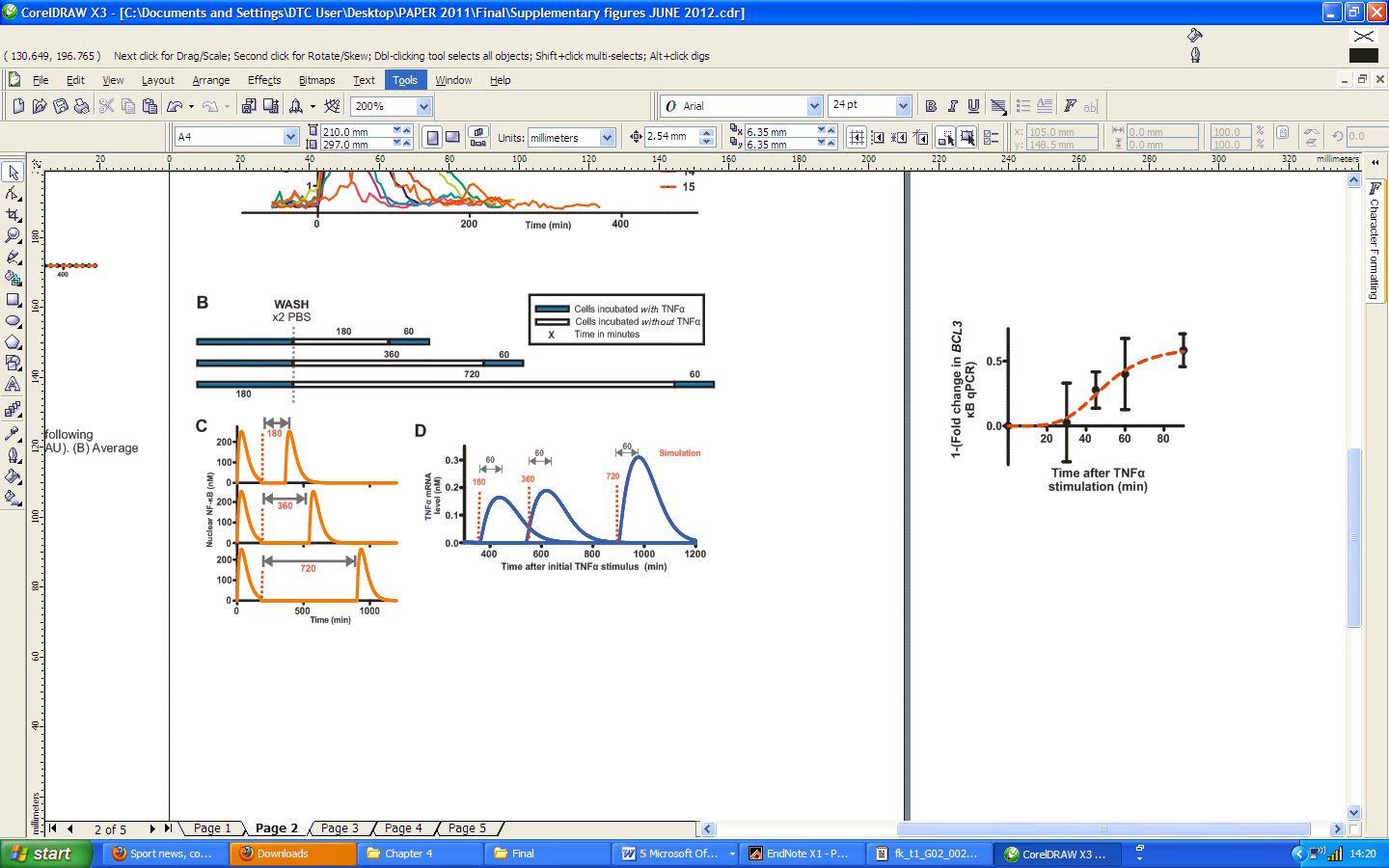


One site – specific binding with Hill slope, R^2^=0.989

Y_MAX_=0.9608; H= 7.347; K_d_=43.16.

**p65 binding at BCL-3 TSS – from Figure 2E**

One site – specific binding with Hill slope, R^2^=0.975

Y_MAX_=1.089; H= 4.473; K_d_=50.99.

**RNA pol. II binding at the BCL-3 TSS – from Figure 2C**

One site – specific binding with Hill slope, R^2^=0.988

Y_MAX_=1.061; H= 5.938; K_d_=55.33.

**BCL-3 mRNA levels – from Figure 1A**

One site – specific binding with Hill slope, R^2^=0.999

Y_MAX_=1189; H= 5.406; K_d_=333.4.
