# Supplementary material for: BCL-3 Attenuation of TNFA Expression Involves an Incoherent Feed-Forward Loop Regulated by Chromatin Structure
Source: PLoS One. 2013 Oct 10;8(10):e77015. doi: 10.1371/journal.pone.0077015 (PMC3794926; doi:10.1371/journal.pone.0077015)
Supplement: Supporting Information S2 — (DOC) [file pone.0077015.s004.doc]

**Supplementary Information S2**

Modelling of the mutual induction of *TNFA* and *BCL3* transcription, incorporating chromatin remodelling at the *BCL3* promoter, was performed using ODE modelling following the scheme outlined in Figure 4B. The equations and parameters used are outlined below.

**Volumes in a fibroblast cell**

The average volume of human fibroblast is considered to be 2,000 μm^3^ with a 5:1 volume ration of cytoplasm:nucleus [1]. Consequently, cytoplasmic and nuclear volumes used are 1,667 and 333 μm^3^ respectively.

**1. Nuclear translocation of NF-κB**

A fixed input of nuclear NF-κB values was used – based on previously measured observations of nuclear p65-dsRed levels in HT1080 cells following TNFα stimulation. Input values were used as averaged from analysis of 15 HT1080 cells; with an equation derived as previously mention in Supplementary Data 1. Maximal nuclear NF-κB concentration is assumed to be 260 nM, as in Ashall et al. [2].

**Equation plotting nuclear levels of NF-κB as a function of time (assuming +TNFα at t=0)**

nNFkB=260*(144*(1-exp(-0.03218*t))-144*(1-exp(-0.03160*t)));

**2. NF-κB induced transcription of the *TNFA* gene**

**Equation : dydt(1) TNFα mRNA**

dydt(1)=k101*((nNFkB./(nNFkB+k102))*(1-(BCL3./(BCL3+k103))))-TNFmRNA*k104;

**Equation for NF-κB/BCL-3 binding at the *TNFA* gene promoter**

Two independent κB sites are assumed to influence *TNFA* transcription – a proximal site which binds NF-κB exclusively and a distal site binding BCL-3 (see main text – Figure 1E). The occurrence of transcription is dependent on the probability of NF-κB being bound at the proximal site and BCL-3 not being bound at the distal site i.e. P(*TNFA* transcription)=P(NF-κB bound at proximal κB site)*(1-P(BCL-3 bound at distal κB site)). Probabilities of binding are dependent on concentrations of the binding factors and dissociation constants for DNA binding. BCL-3 binding has been assumed to occur independently i.e. without p50 or p52 homodimers, which are not considered in this model.

| **Parameter name** | **Description** | **Value** | **Rationale** |
| --- | --- | --- | --- |
| k101 | Maximal rate of *TNFA* transcription | 6.6 x10^-2^ μM.min^-1^ | Calculated* |
| k102 | K_D_ value for NF-κB binding at proximal κB site in *TNFA* promoter | 69 nM | Calculated** |
| k103 | K_D_ value for BCL-3 binding at distal κB site in *TNFA* promoter | 53 nM | Calculated** |
| k104 | Degradation rate for TNFα mRNA | 3.45 x10^-2^ min^-1^ | Measured*** |

* Maximum rate of RNA pol. II initiation assumed to be 33 min^-1^ (based on transcription speed of 55 nt/s and minimal spacing between transcribing polymerases of 100 nt [3] or 66 min^-1^ when both copies of a gene are considered. Transcripts are transported to the cytoplasm (assumed to be instantaneous in relation to the timescale of other processes in this model) where 66 mRNA molecules produced per minute corresponds to 6.6 x10^-2^ nM.s^-1^ (assuming a cytoplasmic volume of 1.67x10^-12^ l).

** The binding of transcription factors such as NF-κB to DNA is dependent on not only the target DNA binding site bound but also physiological factors such as NaCl concentration – as shown by the variation in dissociation constant for p50/p65 heterodmimers binding κB containing DNA sequence in Phelps et al. [4]. NaCl concentrations within nuclei in experiments are considered to be ~100 mM. The dissociation constant for p50/p65 binding at a proximal κB site in the *TNFA* promoter is consequently considered to be 69 nM [4]. The distal κB site within the *TNFA* promoter is, in contrast, a strong palindrome (GGGACCCCCC) – a feature strongly associated with enhanced p50 homodimer (and assumed BCL-3) binding. Relative values for the affinity of classic NF-κB dimers (p50/p65) and BCL-3/p50 homodimer affinity for κB sites is taken from [5]. Here an endogenous IFN-β κB site (GGGxxxxTCC) and palindrome induced mutant version (GGGxxxxCCC) are compared for affinity to p50/p65 heterodimers and p50 homodimers. Relative affinities for the two dimer combinations for the two κB site types from this paper were used to calculate the relative dissociation constant values for BCL-3 (assumed with p50 homodimer) binding a palindromic κB site (such as the distal *TNFA* promoter κB site) and classic NF-κB dimer binding at a non-palindromic κB site (such as the proximal *TNFA* promoter κB site). This ratio was calculated to be 1:1.3 (values measured in molarity); consequently, k103 is valued at 53 nm.

*** Experimentally determined at 20 minutes (see Supplementary Figure S1B).

**3. Histone acetylation: as induced by NF-κB**

To investigate whether NF-κB was involved in histone acetylation at the *BCL3* gene’s TSS, ChIP was again used to determine the relative level of histone 3 acetylation at this site (as in Figure 2C) - using the same primer set – after 90 minutes of TNFα stimulation in cells with and without pre-treatment with the NF-κB inhibitor SN50. A final concentration of 30 ng/ml of SN50 was used (as previously). A significant decrease in histone 3 acetylation was observed in cells pre-treated with SN50, showing a role for NF-κB in mediating this change in histone status (Supplementary Information Figure SI2a). Note that for reasons related to toxicity, SN50 is used under conditions that cause ~50% reduction in target gene mRNA synthesis (Figure 1B), which is consistent with NF-κB playing a dominant role in histone acetylation at the proximal *BCL3* promoter. The occurrence of NF-κB (p65) dependent acetylation has previously been demonstrated [6] and occurs via recruitment of co-activator complexes possessing histone acetylating activity, for example CBP/p300 [7].

**
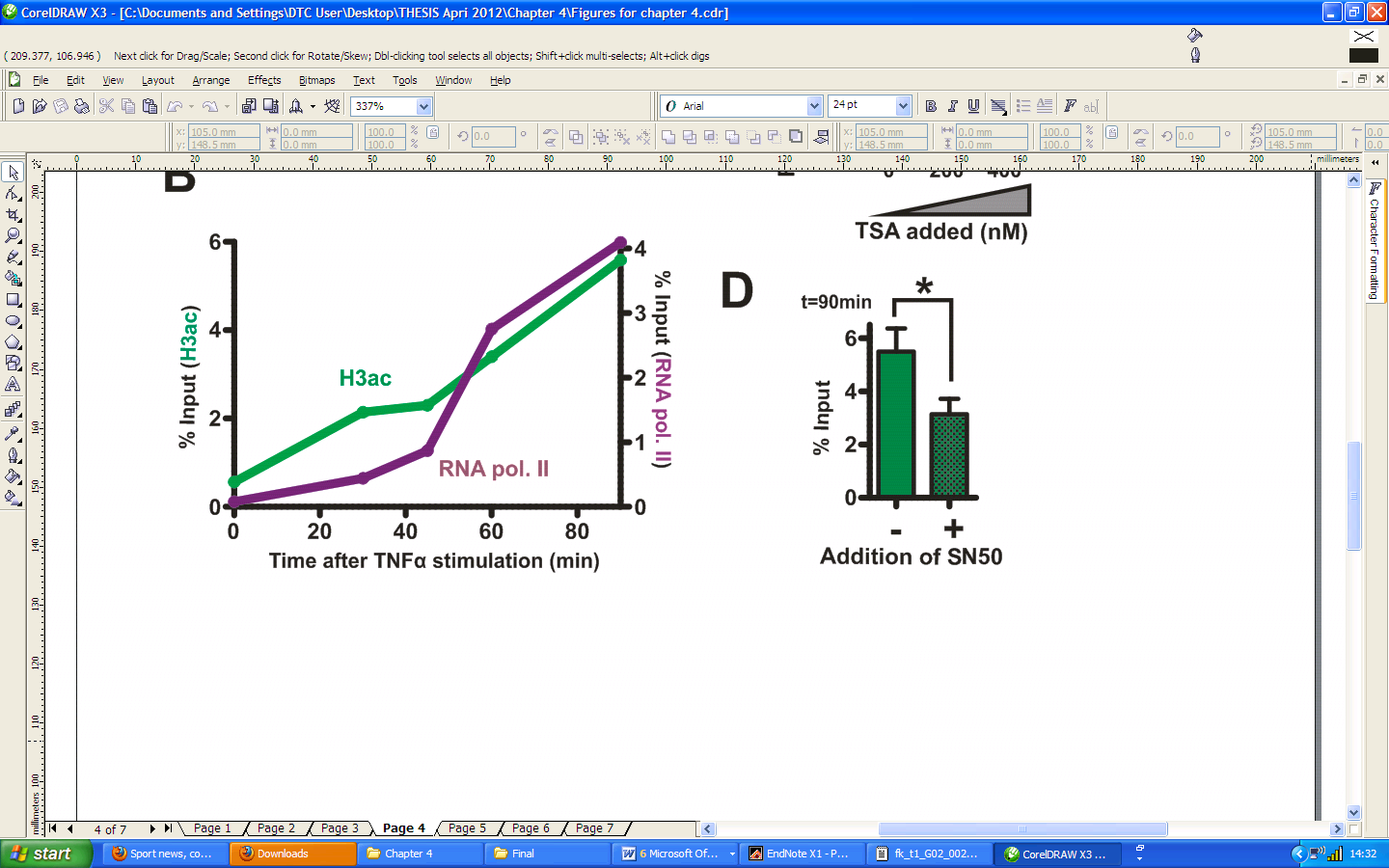
**

**Supplementary Information Figure SI2a. NF-κB signalling mediates TNFα induced histone acetylation at the *BCL3* gene promoter in HT1080 cells.** Cells were induced with TNFα for 90 minutes and Histone 3 acetylation levels at a genomic site proximal to the *BCL3* gene TSS was assayed using ChIP (as before in Figure 2C). Values are given as a percentage of signals from total chromatin Input, as assayed using qPCR. * P<0.05.

**Equation : dydt(2) Histone acetylation level**

dydt(2)=k105*(nNFkB^2./(nNFkB^2+k106^2))-k107*HisAc;

| **Parameter name** | **Description** | **Value** | **Rationale** |
| --- | --- | --- | --- |
| k105 | Rate of histone acetylation | 0.00055 min^-1^ | Fitted* |
| k106 | Value of nNFκB at which half max acetylation rate is induced | 260 nM | Fitted* |
| k107 | Rate of histone deacetylation | 0.00055 min^-1^ | Fitted* |

* The concentration of nuclear NF-κB at which half max acetylation rate is reached, the rates of acetylation and deacetylation plus the Hill coefficient determining the rate of acetylation increase with respect to NF-κB concentration are fitted to the experimentally observed rate of histone 3 acetylation shown in Figure 4A. The fitted values recreate the relative timing and rate of change (increase) in acetylation (see Figure 4C). With regard to the magnitude of response, total concentration of histone acetylated is assumed to be less than or equal to the maximum concentration of histone H3 in the cell. This value is estimated to be ~2x10^-2^ nM - based on four histone 3 molecules (two H3 per nucleosome remodeled in the BCL-3 promoter, 2 gene copies) in a nuclear volume of 3.33x10^-13^ liters.

NF-κB has previously been shown to act as a transcription inducer via recruitment of co-activators such as p300/CBP, p/CAF, and p160 proteins, which posses Histone Acetylase (HAT) activity [8]. Based on the timing of changes during activation (Figure 3) it has been assumed in the model that NF-κB binding at the *BCL3* promoter is required for inducing histone acetylase activity in response to TNFα stimulation. Note that chromatin remodelling at the BCL3 promoter is required at the TSS proximal NF-κB binding site, which is directly responsible for recruiting the transcriptional pre-initiation complex to activate gene expression. NF-κB binding at distal sites within the promoter, and potentially more remote sites that are able to interact with the promoter based on the 3D structure of the active site [9], must contribute to the NF-κB-dependent chromatin remodelling of the proximal promoter following induction of NF-κB signalling.

**4. Chromatin accessibility at the *BCL3* promoter**

**Equation : dydt(3) Chromatin accessibility status**

dydt(3)=k108*(HisAc^3./(HisAc^3+k109^3))-Chr*k110;

‘Chromatin accessibility’ refers to the relative ability of specific transcription factors (NF-κB in this case), general transcription factors and RNAP to bind DNA. In this model, chromatin state is determined by the level of histone acetylation (HisAc) – based on the relationship between the rate of histone H3 acetylation level increase and chromatin accessibility state (Figure 2C). The increase in chromatin accessibility is a specific requirement for binding of NF-κB and RNAP – as supported by the temporal binding of these proteins at the *BCL3* promoter based on measured changes in chromatin accessibility (Figure 4A).

| **Parameter name** | **Description** | **Value** | **Rationale** |
| --- | --- | --- | --- |
| k108 | Rate of chromatin opening | 0.4 min^-1^ | Fitted* |
| k109 | Value of HisAc at which half max rate of chromatin opening is induced | 0.08 nM | Fitted* |
| k110 | Rate of chromatin re-condensation | 0.4 min^-1^ | Fitted* |

* Values fitted to obtain behavior shown in Figure 4A. Values were used such that the magnitude of remodelled chromatin concentration response did not exceed ~1x10^-2^ nM (based on two chromatin molecules – two copies of gene per cell – in a nuclear volume of 3.33x10^-13^ liters).

**5. NF-κB induced *BLC3* transcription**

**Equation : dydt(4) BCL-3 mRNA**

dydt(4)=(Chr^3./(Chr^3+k111^3))*k112*((nNFkB./(nNFkB+k113)*(1-(BCL3./(BCL3+k114)))))-BCL3mRNA*k115;

| **Parameter name** | **Description** | **Value** | **Rationale** |
| --- | --- | --- | --- |
| k111 | Level of chromatin accessibility (‘Chr’) at which half maximal p65 and RNAP access to *BCL3* promoter occurs. | 0.005 nM | Fitted* |
| k112 | Maximal rate of *BCL3* transcription initiation | 6.6 x10^-2^ μM.min^-1^ | As for k101 |
| k113 | K_D_ value for NF-κB binding at κB site in *BCL3* promoter | 69 nM | As for k102 |
| k114 | K_D_ value for BCL-3 binding at κB site to mediate BCL-3 self inhibition** | 53 nM | As for k103 |
| k115 | Degradation rate of *BCL3* mRNA | 4.5x10^-3^ min^-1^ | Measured*** |

* Fitted to replicate timing of BCL-3 transcript induction with respect to the relative level of chromatin accessibility shown in Figure 4A.

** BCL-3 protein has previously been shown to inhibit transcription of its own transcript through binding at an intronic κB site [10]. Dissociation constant is the same as at the *TNFA* promoter (k103).

**** Experimentally determined half life at 154 minutes (see Supplementary Figure S1B).

**6. BCL-3 protein**

**Equation : dydt(5) BCL-3 protein**

dydt(5)=k116*BCL3mRNA-k117*BCL3;

BCL-3 protein is assumed to be available [for DNA binding] immediately following translation, as the rate of diffusion to potential binding sites in the nucleus will be negligible relative to other features of the model. BCL-3 protein has been widely shown to be a predominantly nuclear localised factor in various cell lines [11,12] and this was confirmed in HT1080 cells in this study (Figure 1G).

| **Parameter name** | **Description** | **Value** | **Rationale** |
| --- | --- | --- | --- |
| k116 | Rate of BCL-3 protein synthesis | 3.6 nM/nm of BCL-3 mRNA/min | Calculated* |
| k117 | Rate of BCL-3 protein degradation | 2.4x10^-3^ min^-1^ | Calculated** |

* Maximal rate of translation (3.6 nm/nm of BCL-3 mRNA/min) calculated as in (1), based on a translation rate of 180 amino acids per minute [13] with 9 ribosomes acting on each mRNA - ribosome spacing of 150 nucleotides [14] acting on the ~1400 nt BCL-3 transcript - to produce 3.6 peptides per minute per *BCL3* mRNA molecule in a cytoplasm of volume 1,667 μm^3^.

** Relative rate of BCL-3 protein degradation estimated from [15] – Western blot of relative BCL-3 occurrence at varying lengths of time after cells (Human Embryonic Kidney 293 and Karpas cell lines) had been treated with cyclohexamide. Half-life value calculated at 280 minutes (Supplementary Figure S1C).

**References**

1. Lipniacki T, Paszek P, Brasier AR, Luxon B, Kimmel M (2004) Mathematical model of NF-kappa B regulatory module. J Theor Biol 228: 195-215.

2. Ashall L, Horton CA, Nelson DE, Paszek P, Harper CV, et al. (2009) Pulsatile Stimulation Determines Timing and Specificity of NF-kappa B-Dependent Transcription. Science 324: 242-246.

3. Cheong R, Bergmann A, Werner SL, Regal J, Hoffmann A, et al. (2006) Transient I kappa B kinase activity mediates temporal NF-kappa B dynamics in response to a wide range of tumor necrosis factor-alpha doses. J Biol Chem 281: 2945-2950.

4. Phelps CB, Sengchanthalangsy LL, Malek S, Ghosh G (2000) Mechanism of kappa B DNA binding by Rel/NF-kappa B dimers. J Biol Chem 275: 24392-24399.

5. Fujita T, Nolan GP, Ghosh S, Baltimore D (1992) Independnet modes of transcriptional activaton by the p50-subunit and p65-subunit of NF-kappa-B. Genes Dev 6: 775-787.

6. Boekhoudt GH, Guo Z, Beresford GW, Boss JM (2003) Communication between NF-kappa B and Sp1 controls histone acetylation within the proximal promoter of the monocyte chemoattractant protein 1 gene. J Immunol 170: 4139-4147.

7. Gerritsen ME, Williams AJ, Neish AS, Moore S, Shi Y, et al. (1997) CREB-binding protein p300 are transcriptional coactivators of p65. Proc Natl Acad Sci USA 94: 2927-2932.

8. Gao ZG, Chiao P, Zhang X, Zhang XH, Lazar MA, et al. (2005) Coactivators and corepressors of NF-kappa B in I kappa Beta alpha gene promoter. J Biol Chem 280: 21091-21098.

9. Papantonis A, Kohro T, Baboo S, Larkin JD, Deng BW, et al. (2012) TNF alpha signals through specialized factories where responsive coding and miRNA genes are transcribed. EMBO J 31: 4404-4414.

10. Brocke-Heidrich K, Ge B, Cvijic H, Pfeifer G, Loffler D, et al. (2006) BCL3 is induced by IL-6 via Stat3 binding to intronic enhancer HS4 and represses its own transcription. Oncogene 25: 7297-7304.

11. Nolan GP, Fujita T, Bhatia K, Huppi C, Liou HC, et al. (1993) The BCL-3 protooncogene encodes a nuclear I-kappa-B-like molecule that preferentially interacts with NF-kappa-B p50 and p52 in a phosphorylation-dependent manner. Mol Cell Biol 13: 3557-3566.

12. Zhang Q, Didonato JA, Karin M, McKeithan TW (1994) BCL3 encodes a nuclear protein which can alter the subcellular location of NF-kappa-B proteins. Mol Cell Biol 14: 3915-3926.

13. Siller E, DeZwaan DC, Anderson JF, Freeman BC, Barral JM (2010) Slowing Bacterial Translation Speed Enhances Eukaryotic Protein Folding Efficiency. J Mol Biol 396: 1310-1318.

14. Cataldo L, Mastrangelo MA, Kleene KC (1999) A quantitative sucrose gradient analysis of the translational activity of 18 mRNA species in testes from adult mice. Mol Hum Reprod 5: 206-213.

15. Keutgens A, Shostak K, Close P, Zhang X, Hennuy B, et al. (2010) The Repressing Function of the Oncoprotein BCL-3 Requires CtBP, while Its Polyubiquitination and Degradation Involve the E3 Ligase TBLR1. Mol Cell Biol 30: 4006-4021.
